# High Performance Composite Polymer Electrolytes Doped With Spherical-Like and Honeycomb Structural Li_0.1_Ca_0.9_TiO_3_ Particles

**DOI:** 10.3389/fchem.2018.00525

**Published:** 2018-10-25

**Authors:** Wei Xiao, Zhiyan Wang, Chang Miao, Ping Mei, Yan Zhang, Xuemin Yan, Minglei Tian, Yu Jiang, Jingjing Liu

**Affiliations:** ^1^College of Chemistry and Environmental Engineering, Yangtze University, Jingzhou, China; ^2^Environmental Monitoring Department, Changsha Environmental Protection College, Changsha, China

**Keywords:** Li_0.1_Ca_0.9_TiO_3_, composite polymer electrolyte, spherical-like, honeycomb, lithium ion battery

## Abstract

The spherical-like and honeycomb structural Li_0.1_Ca_0.9_TiO_3_ particles are prepared by spray drying combined with following calcination confirmed by X-ray diffraction (XRD) and scanning electron microscopy (SEM) with energy dispersive X-ray spectrometer (EDS). The poly (vinylidene fluoride-*co*-hexafluoropropylene) (P(VDF-HFP))-based composite polymer electrolytes (CPEs) modified with the particles are fabricated by phase inversion and activation processes. The characterization results show that the as-prepared CPE membranes possess the smoothest surface and most abundant micropores with the lowest crystallinity with adding the particles into the polymer matrix, which results in high ionic conductivity (3.947 mS cm^−1^) and lithium ion transference number (0.4962) at ambient temperature. The interfacial resistance can be quickly stabilized at 508 Ω after 5 days storage and the electrochemical working window is up to 5.2 V. Moreover, the mechanical strength of the membranes gains significant improvement without lowering the ionic conductivity. Furthermore, the assembled coin cell can also deliver high discharge specific capacity and preserve steady cycle performance at different current densities. Those outstanding properties may be ascribed to the distinctive structure of the tailored spherical-like and honeycomb structural Li_0.1_Ca_0.9_TiO_3_ particles, which can guarantee the desirable CPEs as a new promising candidate for the polymer electrolyte.

## Introduction

At present, more and more attentions have been invested into the polymer electrolytes in the field of the lithium ion battery, which can not only realize the flexible assembly of the packed battery, but also effectively prevent the explosion and spontaneous combustion incidents of the assembled battery (Croce et al., 1998; Song et al., 1999; Scrosati, 2000; Wang et al., 2014; Wu et al., 2017b; He et al., 2018a). However, the solid polymer electrolytes (SPEs) are excluded from the practical use because of their quite low ionic conductivity of the assembled lithium ion battery (Murata et al., 2000; Xie et al., 2012; Kim et al., 2015; Zhang H. et al., 2017; Wu et al., 2018). The ionic conductivity at room temperature can gain noticeable improvement when the polymer electrolyte membranes adsorb some liquid electrolytes to form the novel gel polymer electrolytes (GPEs), which are mainly composed of the polymer matrix and the entrapped liquid electrolytes (Zhang et al., 2007; Hu et al., 2015; Zhang M. Y. et al., 2017; Wang et al., 2018c). However, the poor mechanical strength of the swollen GPEs cannot well maintain the separation between the positive and negative active materials during the long-term Li^+^ insertion-extraction behaviors (Rao et al., 2012; Liu et al., 2017; Luo et al., 2017). Therefore, some countermeasures have been taken to circumvent the obstacles. Adding inert inorganic nano-particles, such as TiO_2_ (Wu et al., 2011; Chen et al., 2015; Zhang et al., 2015; He et al., 2018b), Al_2_O_3_ (Lee et al., 2014; Liang et al., 2015; Wu et al., 2017a), and SiO_2_ (Park et al., 2010; Li et al., 2013; Huang et al., 2017; Shim et al., 2017), into the polymer matrix to fabricate the composite polymer electrolytes (CPEs) has been ever proved to be an available and appealing approach to balance the contradiction between the ionic conductivity and mechanical strength at room temperature (Costa et al., 2013; Wang et al., 2018b). However, the poor dispersibility of the added nano-inorganic particles into the polymer substrate is also the barrier for the as-fabricated CPEs. Perovskite structural ABO_3_ particles doped with metal ions can produce more vacancies, which can facilitate the ions transfer to enhance the ionic conductivity as well as improving the mechanical strength (Kay and Bailey, 1957; Mather et al., 2007). In the work, spherical-like and honeycomb perovskite structural Li_0.1_Ca_0.9_TiO_3_ particles are successfully synthesized by spray drying combined with sintering, and the desirable CPEs are prepared by traditional phase inversion method. The corresponding electrochemical and battery performance of the CPEs are also carefully studied.

## Experimental

### Preparation of the Li_0.1_Ca_0.9_TiO_3_ powders

The spherical-like and honeycomb structural Li_0.1_Ca_0.9_TiO_3_ particles are prepared by spray drying combined with following calcination. At first, stoichiometric calcium acetate and lithium acetate are successively added into deionized water to form solution A, and the mixture of tetrabutyl titanate, acetylacetone, and absolute ethyl alcohol with the volume ratio of 4:1:4 is dropwise added into the solution A to obtain homogeneous solution B with continuous agitation at 40°C for 6 h. Secondly, the solution B is pumped into the spray drier machine (SD-2500) by peristaltic pump at 1,500 mL h^−1^ and atomized at 180°C with atomizing pressure of 0.2 MPa to product the precursor powders with conditions similar to the scheme (Yang et al., 2014; Su et al., 2018). The target product Li_0.1_Ca_0.9_TiO_3_ particles can be gained after the precursor powders are further calcined in air at 700°C for 6 h. All chemicals are purchased from Macklin Biochemical Co., Ltd. with analytical grade and used without any further purification.

### Fabrication of the CPEs

The fabrication processes of the CPEs can be concisely summarized as fabrication and activation of the polymer electrolyte membranes. At first, a certain content of Li_0.1_Ca_0.9_TiO_3_, urea and poly (vinylidene fluoride-*co*-hexafluoropropylene) P(VDF-HFP) (Atofina, Kynar Flex, 12 wt.% HFP) are successively fed into the N, N-dimethylformamide (DMF) solvent to yield uniform coating solution under vigorous agitation at room temperature, in which Li_0.1_Ca_0.9_TiO_3_, urea and P(VDF-HFP) powders are used as dopant, pore-forming agent and polymer matrix, respectively, and the weight ratio is maintained at about 5:1:100. After that, the homogeneous casting solution is cast onto a pre-cleaned glass substrate with a doctor blade to prepare the wet membranes. Then, the wet membranes are immersed into deionized water for 12 h at room temperature to thoroughly fulfill phase transfer and the free-standing CPE membranes can be obtained by being dried for 24 h at 60°C to remove the residual solvent and then cut into a disk with a diameter about 16 mm for use (Wang et al., 2018b; Xiao et al., 2018). Secondly, the desirable CPEs can be acquired after immersing the as-prepared CPE membranes into 1.0 M LiPF_6_-EMC/EC/DMC (1:1:1, *v/v/v*) provided by Dongguan Shanshan Battery Materials Co., Ltd. for 0.5 h, which are labeled as CPE-LCT for convenience. The polymer electrolytes doped with commercial CaTiO_3_ and without inorganic particles are also fabricated using the same method for comparison and named as CPE-CT and CPE-0, respectively.

### Properties characterization

Scanning electron microscopy (SEM, JSM6301F) equipped with an energy dispersive X-ray spectrometer (EDS) and X-ray diffraction (XRD, Rint-2000) are used to investigate the micro-structure, chemical composition, and physical phase constitution of the as-prepared Li_0.1_Ca_0.9_TiO_3_ particles. The surface morphology and crystallinity of the as-fabricated CPE membranes are identified by SEM and XRD, respectively. The porosity (*P*) and liquid uptake rate (*A*) of the as-prepared CPE membranes are calculated according to the our previous work in the following Equations (1, 2), respectively, in which ρ_*a*_ and ρ_*b*_ are the density of *n*-butanol and the dry CPE membrane, *m*_*a*_ (*w*_1_) and *m*_*b*_ (*w*_0_) are the mass (weight) of the membranes with and without *n*-butanol, respectively (Xiao et al., 2016). The mechanical tensile strength (*T*) of the as-prepared CPE membranes is performed on an electronic universal testing machine (INSTRON-5500) at a crosshead speed of 100 mm min^−1^ at room temperature, which refers to the literature (Wang et al., 2018a). To reveal the electrochemical performance, the as-fabricated CPEs are packed in the simulated battery in the following three types. The first type is that the CPE is assembled between two stainless steel (SS) electrodes, i.e., SS/CPE/SS, which is employed to test the ionic conductivity at various temperatures (293–363 K) by electrochemical impedance spectroscopy (EIS) on the CHI660E electrochemical workstation with frequency range 10^5^-1 Hz. The ionic conductivity can be calculated from the bulk resistance (*R*) according to Equation (3), where *l* is the thickness of the CPE, *S* is the effective contact area, *R* is obtained from the Nyquist impedance plots (Xiao et al., 2016). The second one is that the CPE is assembled between a lithium disk electrode and a SS electrode, i.e., Li/CPE/SS, which is utilized to evaluate the electrochemical working window of the CPE by the EIS from 2.50 to 6.65 V with a scanning rate of 5 mV s^−1^. The last one is that the CPE is packed between two lithium disk electrodes, i.e., Li/CPE/Li, which is used to monitor the interfacial resistance changes with different storage times at 30°C. The lithium ion transference number introduced by the literature (Bruce and Vincent, 1987; Xiao et al., 2016) of the symmetrical Li/CPE/Li cells is measured by the combination of Dc polarization and EIS techniques. The corresponding value can be calculated according to the reference in the following Equation (4), in which *I*_0_ and *I*_*ss*_ are the initial and the steady current, *R*_0_ and *R*_*ss*_ are the initial interfacial and the steady-state resistance, respectively, and Δ*V* is the potential difference (Xiao et al., 2016).

(1) $$\begin{array}{rcl} P\% & = & \frac{m_{a}/\rho_{a}}{\left(m_{a}/\rho_{a}\right)+\left(m_{b}/\rho_{b}\right)}\times 100\% \end{array}$$

(2) $$\begin{array}{rcl} A\% & = & \frac{w_{1}-w_{0}}{w_{0}}\times 100\% \end{array}$$

(3) $$\begin{array}{rcl} \sigma & = & l/(R\cdot S) \end{array}$$

(4) $$\begin{array}{rcl} T_{Li^{+}} & = & \frac{I_{ss}\left(\Delta V-R_{ss}I_{0}\right)}{I_{0}\left(\Delta V-R_{0}I_{ss}\right)} \end{array}$$

The charge-discharge cycling performance of the assembled Li/CPEs/LiCoO_2_ coin cells are performed on a Land Battery Test System (Wuhan Land Electronic Corporation, China) at different current densities (0.1, 0.2, 0.5, 1.0, and 2.0 *C*) with cut-off voltages of 2.75–4.25 V at room temperature, in which the working electrode is fabricated by casting the mixed slurry of 80 wt.% LiCoO_2_ as active material, 10 wt.% acetylene black as conductive agent and 10 wt.% polyvinylidene fluoride as binder onto aluminum foils with mass loading of 2–3 mg cm^−2^, and the thickness of the as-fabricated CPEs is about 120 μm (Xiao et al., 2016, 2018; Wang et al., 2018b).

## Results and discussion

**Figure 1:**
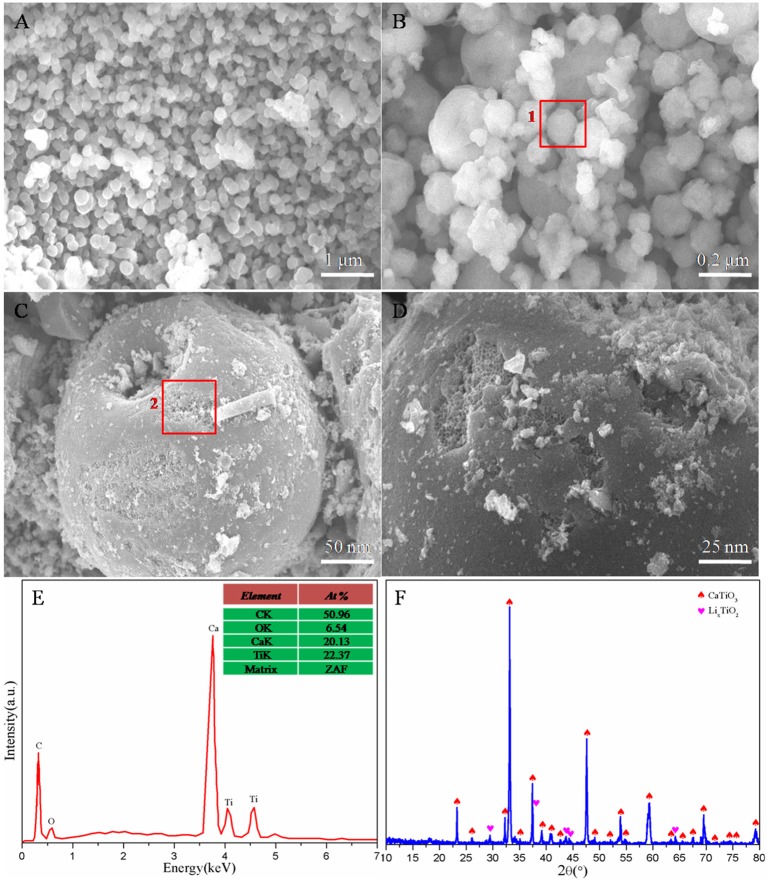
SEM **(A–D)**, EDS **(E)**, and XRD **(F)** images of the as-prepared Li_0.1_Ca_0.9_TiO_3_ particles.

Figure 1 displays the micro-structure, chemical composition, and physical phase constitution of the as-prepared Li_0.1_Ca_0.9_TiO_3_ particles. It can be clearly seen from Figures 1A,B that the SEM images present homo-dispersed spherical-like particles with the average diameter of 250 nm, which can be confirmed by the enlarged SEM image of the area 1 demonstrated in Figure 1C. It is worth noting that the Li_0.1_Ca_0.9_TiO_3_ particles show coarse and damaged surface in Figure 1C. It can be observed from the enlarged SEM image of the area 2 demonstrated in Figure 1D that the coarse and damaged surface of the as-prepared particles has abundant honeycomb structures. Therefore, we can speculate that the inside of the as-prepared Li_0.1_Ca_0.9_TiO_3_ particles may possess well-developed interpenetrating porous network structures. Those results indicate that the as-prepared Li_0.1_Ca_0.9_TiO_3_ particles can not only absorb and retain more liquid electrolytes, but also provide more extra passages for lithium ions transfer, which can markedly improve the battery performance. Figure 1E demonstrates the EDS results of the selected area 2 in Figure 1C. It can be obviously found that the as-synthesized nano-inorganic fillers are composed of Ca, Ti, and O elements and the percentage of Ca and Ti atoms is about 0.902:1, which is in accord with the predetermined stoichiometric ratio. The Li element cannot be detected in the EDS plots because of its less content and lighter weight (Sabiha et al., 2017). As demonstrated in Figure 1F, the XRD patterns of the as-synthesized Li_0.1_Ca_0.9_TiO_3_ particles are well indexed to the perovskite structural CaTiO_3_ (JCPDS#82-0228), which would benefit the lithium ions transfer. Moreover, few impurities corresponding to Li_2_TiO_3_ (JCPDS#51-0050) can be detected due to the excessive added lithium salt. Those results suggest that the spherical-like and honeycomb structural Li_0.1_Ca_0.9_TiO_3_ particles are successfully synthesized by spray drying combined with following calcination.

**Figure 2:**
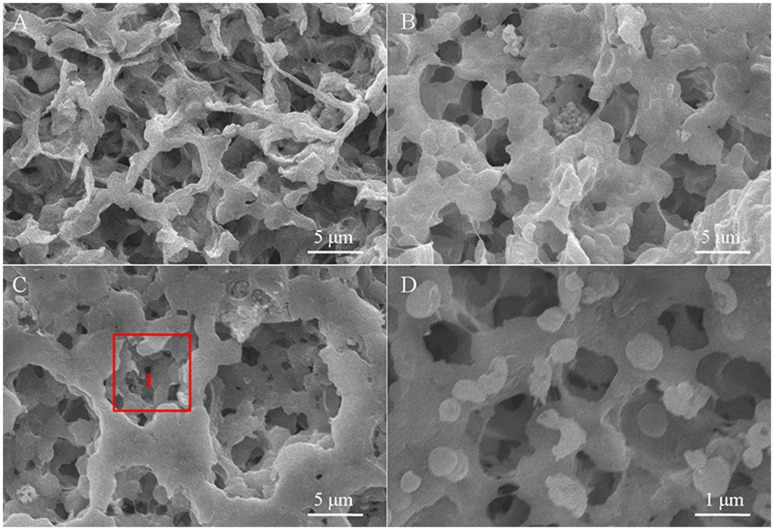
SEM images of the different CPE membranes **(A**, CPE-0; **B**, CPE-CT; **C**, CPE-LCT) and the partial enlarged SEM image of area 1 **(D)**.

The SEM images of the polymer electrolyte membranes doped with different kinds of inorganic particles are displayed in Figure 2. The surface of the as-fabricated CPE membranes presents significant differences with adding the inorganic particles into the polymer substrate, where the surface gets smoother and the pore-size distribution becomes more uniform compared with the pure polymer electrolyte membrane. Although the added particles are obviously discovered in the CPE-CT and CPE-LCT membranes, few aggregations appear on the surface of the CPE-CT membrane displayed in Figure 2B, which can be ascribed to the high specific surface energy of the added commercial nano-CaTiO_3_ powders. The as-synthesized Li_0.1_Ca_0.9_TiO_3_ particles can be uniformly dispersed into the polymer matrix demonstrated in Figure 2C because of their unique spherical-like and honeycomb structure, which can be confirmed by the partial enlarged SEM image of the selected area 1 displayed in Figure 2D. The added Li_0.1_Ca_0.9_TiO_3_ particles well dispersed into the polymer electrolyte membranes with abundant micro-pore structures can not only enhance the mechanical properties by providing more physical crosslinking sites, but also improve the ionic conductivity by retaining more liquid electrolytes at the same time, which can be proved by the results listed in Table 1. As listed in Table 1, the porosity, uptake rate and tensile strength of the as-prepared CPE membranes gain remarkable improvements with doping the inorganic fillers into the polymer substrate, in which the values of the CPE-LCT membrane can reach to the highest value of about 92.73, 168.5%, and 27.32 Mpa, respectively. Clearly, these results can be mainly to the unique spherical-like and honeycomb structural of the added Li_0.1_Ca_0.9_TiO_3_ particles, in which the external honeycomb structure can provide more space to store liquid electrolytes and transfer lithium ions and the inert spherical-like structure can maintain the mechanical properties of the membranes.

**Table 1:** Porosity, uptake rate, tensile strength, ionic conductivity, activation energy, and lithium ion transference number of different CPE membranes at room temperature.

	***P*/%**	***A*/%**	***T*/MPa**	**σ/mS cm^−1^**	***E_*a*_*/KJ mol^−1^**	***T_*Li*+_***
CPE-0	75.43	89.99	18.84	1.954	7.625	0.1623
CPE-CT	80.62	126.3	26.87	2.818	6.058	0.2764
CPE-LCT	92.73	168.5	27.32	3.947	4.019	0.4962

**Figure 3:**
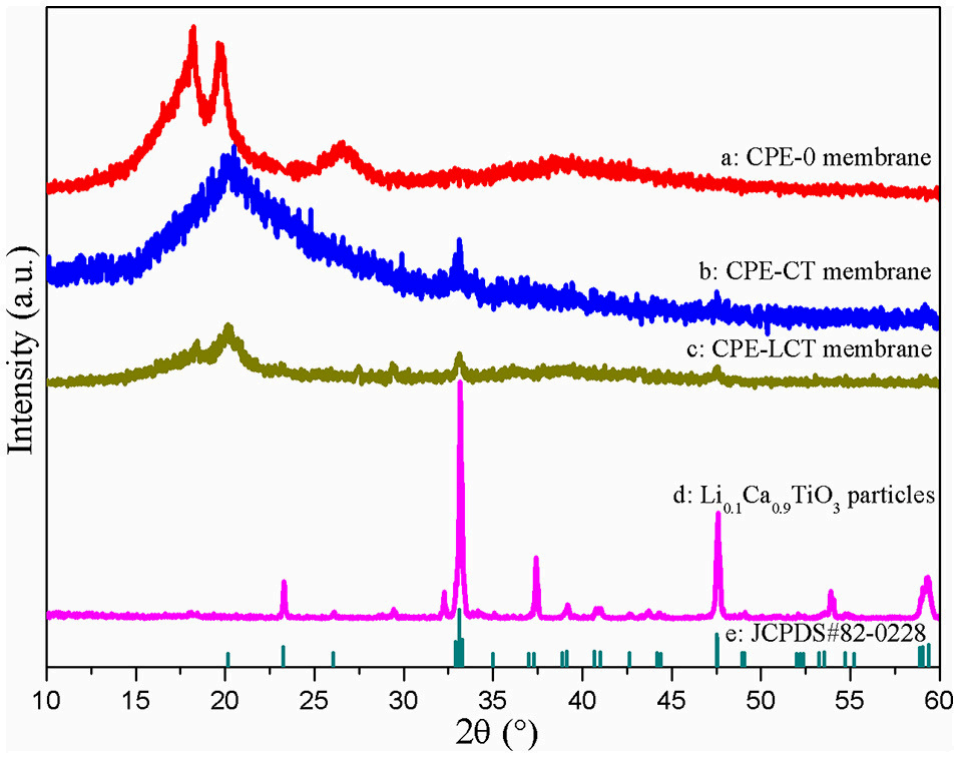
XRD patterns of the as-prepared Li_0.1_Ca_0.9_TiO_3_ particles and different CPE membranes.

Figure 3 demonstrates the XRD patterns of the as-prepared Li_0.1_Ca_0.9_TiO_3_ powders and different CPE membranes. It can be observed from Figure 3 that the XRD patterns of the doped CPE membranes present distinct differences compared to the pristine P(VDF-HFP) one, in which the characteristic diffraction peaks of the polymer matrix P(VDF-HFP) at around 18.3 and 26.6° disappear and the one at 20.0° gets weak. Those results suggest that adding inorganic particles into the polymer matrix can impair the crystallinity of the polymer electrolyte membranes and release more amorphous areas for lithium ions transfer, which are the same as our previous results (Wang et al., 2018c). What is noteworthy is that the CPE-LCT membrane demonstrated in Figure 3 presents the weakest intensity, and we have reasons to speculate that the CPE-LCT may have high ionic conductivity at room temperature, which can be partly attributed to the Lewis acid-base interactions between the doped particles and the polymer chains, and partly to the increasing amorphous areas (Xiao et al., 2014a). Moreover, the new peak can be observed at 32.6° in the doped CPE membranes, which can be ascribed to the characteristic diffraction peak of the as-synthesized perovskite Li_0.1_Ca_0.9_TiO_3_ inorganic particles that are well indexed to the JCPDS#82-0228.

**Figure 4:**
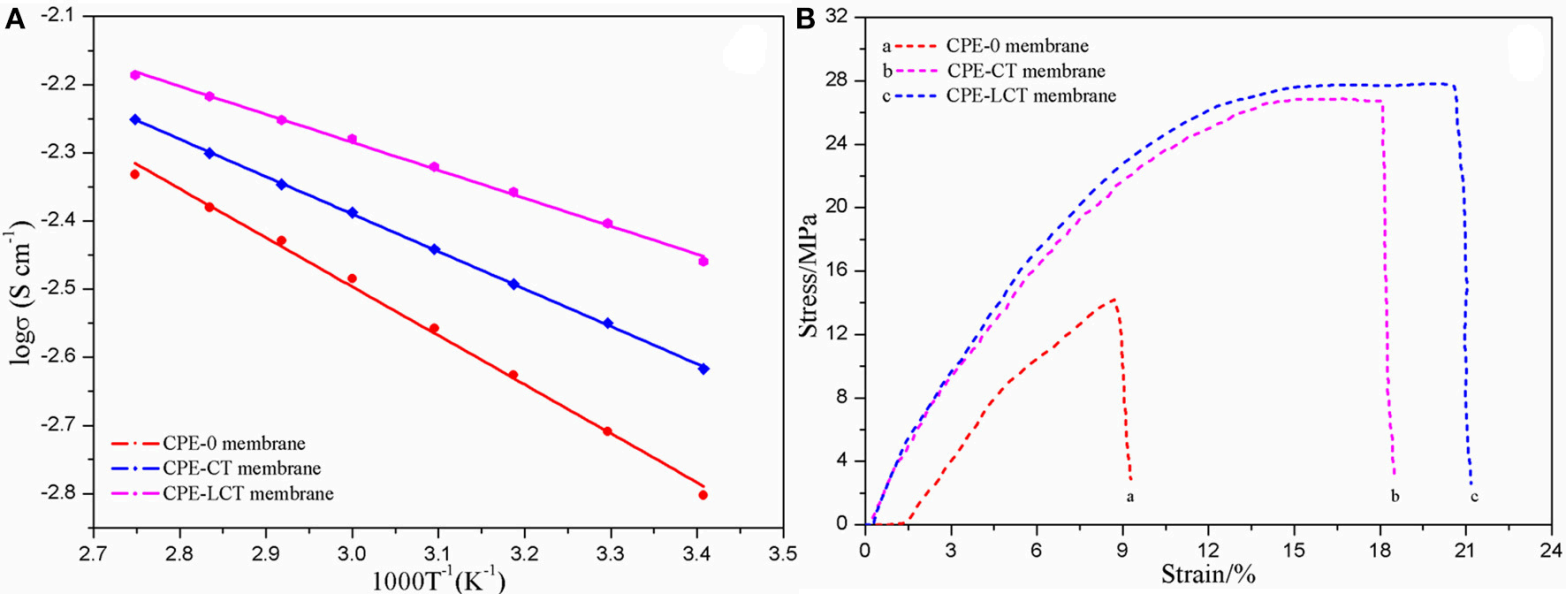
Fitting curves on reciprocal temperature dependence of ionic conductivity **(A)** and the relation of stress and strain **(B)** of the different CPE membranes.

To further investigate the ionic conductivity of the as-prepared CPEs, we have tested the EIS pots of the assembled SS/CPE/SS battery at different temperatures and the corresponding curves on reciprocal temperature dependence of ionic conductivity are demonstrated in Figure 4A. It can be distinctly discovered that the curve of the SS/CPE-LCT/SS battery is always above the other ones, which indicates the CPE doped with Li_0.1_Ca_0.9_TiO_3_ powders may possess the highest ionic conductivity. The ionic conductivity at room temperature of the various CPEs is demonstrated in Table 1, in which the value of the CPE-0, CPE-CT, and CPE-LCT is about 1.954, 2.818, and 3.947 mS cm^−1^, respectively. As displayed in Figure 4A, the plots of the reciprocal temperature vs. ionic conductivity can well fitted by the Arrhenius equation, which indicates that the lithium ions transfer in the CPEs system is mainly attributed to the free diffusions rather than the hopping movements elaborated in our previous work (Xiao et al., 2014b, 2016, 2018; Wang et al., 2018a). The lithium ions can easily migrate from the inside of the spherical-like and honeycomb structural Li_0.1_Ca_0.9_TiO_3_ particles to the interface between the electrodes and the as-prepared CPEs to participate into the intercalation and deintercalation processes in the CPEs system. The corresponding threshold energy of the lithium ions transfer for the different CPEs is calculated and listed in Table 1. As demonstrated in Table 1, the CPE-LCT has the lowest activation energy of about 4.019 KJ mol^−1^. The highest ionic conductivity with the lowest activation energy at room temperature of the as-prepared CPE-LCT can be explained as the following reasons. Firstly, the appropriate size and uniform distribution of the micropores in the as-fabricated CPE-LCT membrane can adsorb and hold more liquid electrolytes, which can significantly promote the ionic conductivity of the system. Secondly, the Lewis acid-base interactions between the added inorganic particles and the polymer matrix can release more amorphous areas for lithium ions transfer (Xiao et al., 2018). Moreover, the lithium ions can migrate in the extra passageways provided by the abundant and interpenetrated micropores in the as-prepared honeycomb structural Li_0.1_Ca_0.9_TiO_3_ particles. The as-synthesized Li_0.1_Ca_0.9_TiO_3_ particles can not only dramatically enhance the ionic conductivity, but also improve the mechanical strength of the corresponding doped CPE membranes at the same time, which are principally ascribed to the excellent mechanical performance of the unique spherical-like and honeycomb structure. As displayed in Figure 4B, the mechanical strength is sharply enhanced from 18.84 MPa of the CPE-0 membrane, to 26.87 MPa of the CPE-CT one, and to 27.32 MPa of the CPE-LCT one. Moreover, the elongations of the CPE-CT and CPE-LCT membranes are also apparently greater than the one of the CPE-0 membrane, in which the elongation at the critical point is about 19.7 and 21.4% for the CPE-CT and CPE-LCT membranes, respectively, and the value is only 13.1% for the pure one. Those results can be ascribed to the more crosslinking sites provided by the doped particles, which can well improve the flexibility of the as-prepared polymer electrolyte membranes to enhance the battery performance.

**Figure 5:**
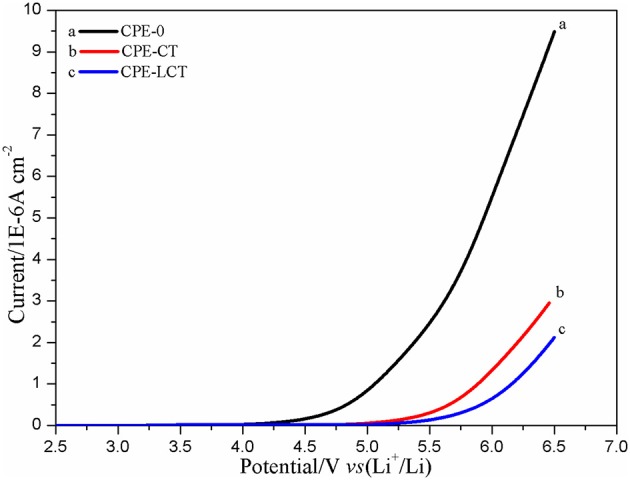
LSV plots of the different CPE membranes.

**Figure 6:**
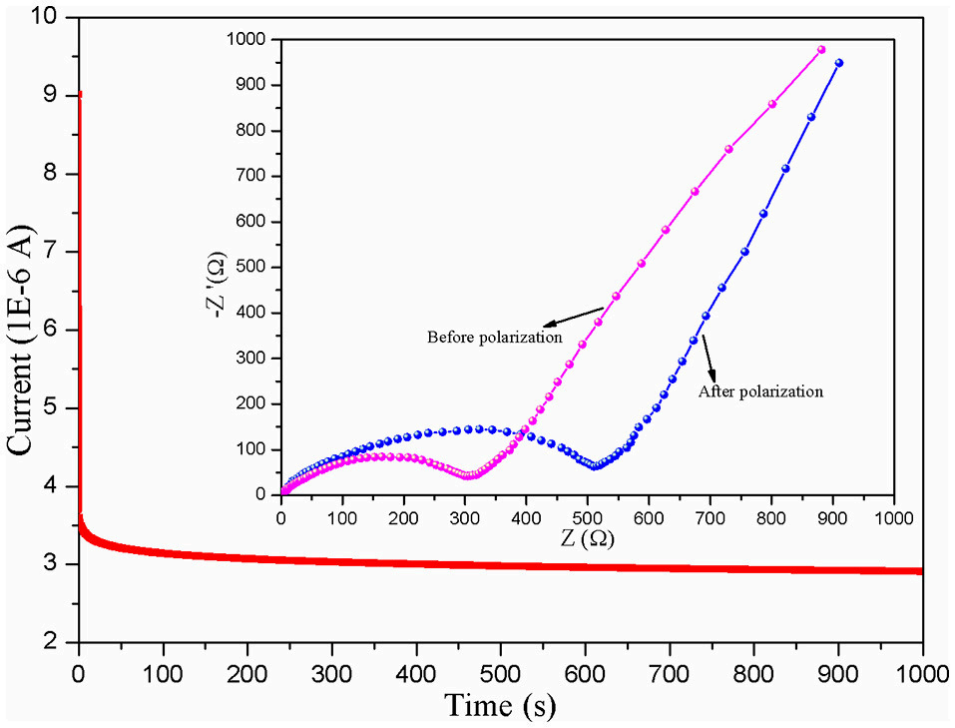
Dc polarization plots of the assembled Li/CPE-LCT/Li battery and the insert are Nyquist plots before and after polarization.

Figure 5 displays the LSV plots of the different CPE membranes. The electrochemical working window of the assembled SS/CPEs/Li cell with inorganic particles is much wider than the one of the cell with the CPE-0, in which the value of the steady electrochemical working window is about 4.3, 5.0, and 5.2 V for the CPE-0, CPE-CT, and CPE-LCT membrane, respectively. The dramatically enhanced working voltage of the doped CPE membranes can be resulted from the gelatinization of the entrapped liquid electrolytes and the added inert nano-particles. The gelation enhances the stability of the CPE membranes due to the interactions between the entrapped liquid electrolytes and the polymer matrix, which can restrict the free movement and the activity of the liquid electrolytes in the as-prepared CPEs (Wang et al., 2018a). The results can be ulteriorly reinforced by adding the as-synthesized Li_0.1_Ca_0.9_TiO_3_ particles into the polymer matrix because of their intrinsic chemical inertness and unique spherical-like and honeycomb structure. Based on the analysis mentioned above, it is creditable to conclude that the CPEs doped with the as-prepared spherical-like and honeycomb structural Li_0.1_Ca_0.9_TiO_3_ particles can provide high lithium ion transference number. Therefore, we design experiments to calculate the lithium ion transference number. The Dc polarization plots of the assembled Li/CPE-LCT/Li battery are demonstrated in Figure 6 and the corresponding results calculated to Equation (4) are displayed in Table 1. It can be observed from Table 1 that the lithium ion transference number increases from 0.1623 of the CPE-0, to 0.2764 and 0.4962 of the CPE-CT and CPE-LCT, respectively, which can perfectly verify the above suppositions. Those results can be mainly ascribed to the added spherical-like and honeycomb structural Li_0.1_Ca_0.9_TiO_3_ particles, which can not only provide more passageways for ion transfer, but also release more lithium ions to increase the carrier concentration.

**Figure 7:**
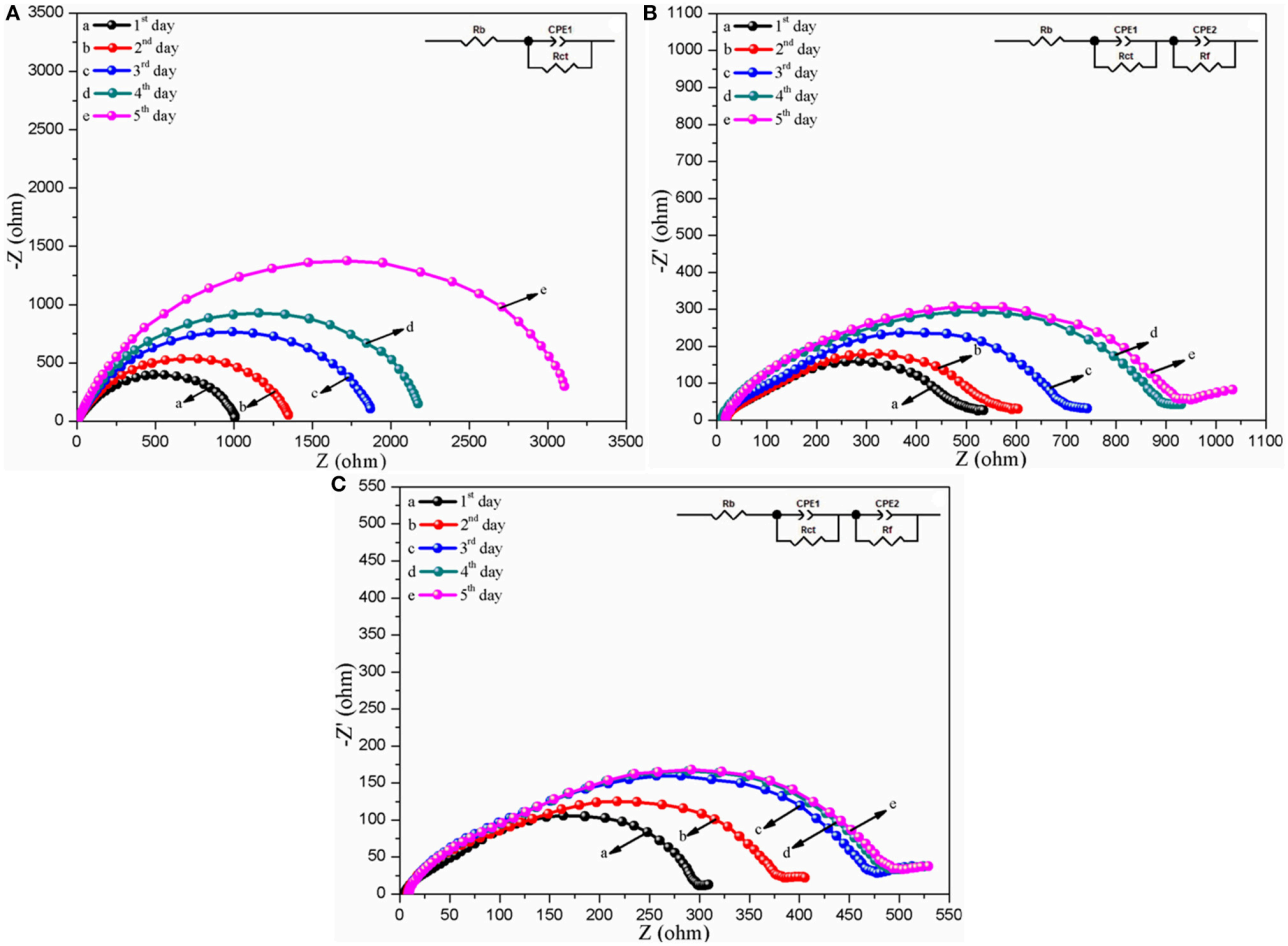
Nyquist plots of the assembled Li/CPEs/Li simulated cells with different CPEs for various storage times at 30°C **(A**, CPE-0; **B**, CPE-CT; **C**, CPE-LCT), where the insert shows the corresponding equivalent circuit.

Interface stability is a key parameter for the practical polymer electrolytes in the lithium ion battery because it can seriously exert an effect on the initial charge-discharge and rate performance of the as-assembled battery (Zhang M. Y. et al., 2017). Therefore, the EIS variations of the Li/CPEs/Li simulated cells with the CPEs for different storage times at 30°C are investigated and the corresponding plots are displayed in Figure 7. It can be easily found from Figure 7 that the shape of the curves of the assembled Li/CPEs/Li simulated cells with three kinds of polymer electrolytes show significant differences, in which the plots of the Li/CPE-CT/Li and Li/CPE-LCT/Li simulated batteries are composed of a semicircle at high and medium frequency range originated from the bulk resistance (*R*_*b*_) of polymer electrolyte and a compressed arc at low frequency range derived from the interfacial resistance (*R*_*i*_), while the one of the Li/CPE-0/Li simulated cell simply contains a semicircle in the whole tested frequency range. Those differences can be mainly ascribed to the different interfacial performance of the as-assembled simulated cells with different CPEs. To further reveal the mechanism of the interfacial performance, the EIS plots are fitted by the corresponding equivalent circuit displayed in the insert for the each plot, in which we decompose interfacial resistance (*R*_*i*_) into interfacial reaction resistance (*R*_*ct*_) and the resistance of charge transfer (*R*_*f*_) in the electronic double-layer for convenience (Xiao et al., 2012). Apparently, the fitting curves are well-matched with the experimental data for the different CPEs shown in Figure 7. The *R*_*b*_ value in each plot keeps constant and the *R*_*ct*_ value increases firstly and then reaches a stable value, in which the *R*_*ct*_ values of the CPE-CT and CPE-LCT can stabilize at around 943 and 508 Ω after being monitored for 5 days at room temperature, while the *R*_*ct*_ value of the CPE-0 keeps growing. The results suggest that the battery assembled with the CPEs may present excellent interfacial performance, especially with the CPE-LCT, which can be mainly ascribed to the added spherical-like and honeycomb structural Li_0.1_Ca_0.9_TiO_3_ particles that can markedly improve the compatibility between the electrodes and the as-prepared CPEs by entrapping any impurities such as water and trace organic solvent to inhibit the destructive reaction on the electrodes (Xiao et al., 2016).

**Figure 8:**
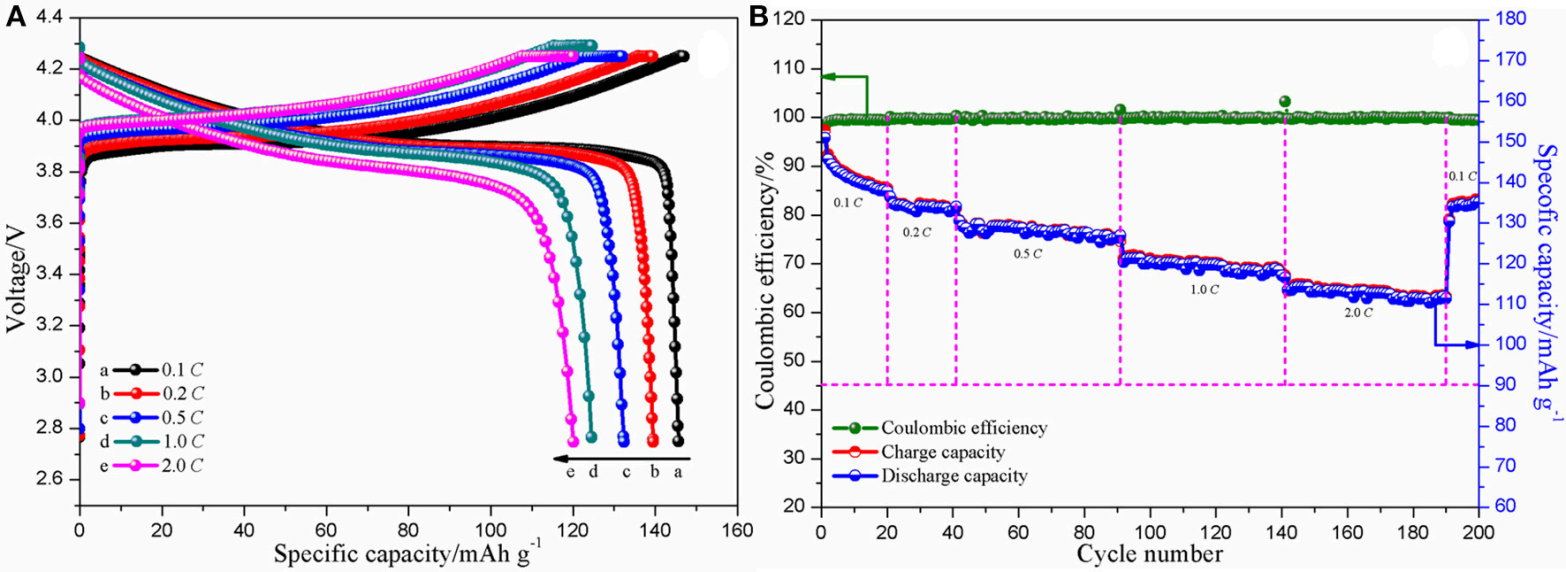
Initial charge-discharge **(A)** and cycle and coulombic efficiency **(B)** curves of the assembled Li/CPE-LCT/LiCoO_2_ coin cell at different current densities.

Initial charge-discharge curves of the assembled Li/CPE-LCT/LiCoO_2_ cell at different current densities (0.1, 0.2, 0.5, 1.0, and 2.0 *C*) are shown in Figure 8A. There is no difference between the cells assembled with the as-prepared CPE membranes and the commercial polyolefin membrane in terms of the shape of the curves, which suggests that the charge-discharge mechanism of the battery cannot be affected when the as-fabricated CPEs are assembled into the coin cell. As found in Figure 8A, the discharge specific capacity slowly declines from 145.7 mAh g^−1^ at 0.1 *C*, to 139.5 and 132.4 mAh g^−1^ at 0.2 and 0.5 *C*, respectively, and maintains at 116.8 mAh g^−1^ even at 2.0 *C*, which indicate that the battery assembled with the CPE-LCT presents excellent charge-discharge performance. Figure 8B demonstrates the cycle and coulombic efficiency curves of the Li/CPE-LCT/LiCoO_2_ coin cell at different current densities (0.1, 0.2, 0.5, 1.0, and 2.0 *C*). It can be obviously observed that the coulombic efficiency of the assembled battery is ~100% except for the first two cycles, which can be mainly ascribed to the formation of steady solid electrolyte interphase films on the surface of the electrodes (Reddy et al., 2013). The discharge capacity gently fades with increasing the current density during the repetitive cycles, in which the battery can deliver about 125.7 mAh g^−1^ discharge specific capacity at 1.0 *C* after 100 cycles with 86.32% capacity retention ratio. Moreover, the discharge specific capacity of the battery can rebound back to 136.4 mAh g^−1^ when the current density drops down to 0.1 *C* again after 190 cycles. Those results suggest that the Li/CPE-LCT/LiCoO_2_ coin cell shows outstanding rate cycle performance with approaching 100% coulombic efficiency.

## Conclusions

The CPEs doped with the spherical-like and honeycomb structural Li_0.1_Ca_0.9_TiO_3_ particles synthesized by spray drying combined with following calcination are successfully fabricated by the classical phase inversion processes. The investigation results show that the CPEs present excellent physicochemical properties, such as improved ionic conductivity and lithium ion transference number, enhanced interfacial stability, and mechanical strength and so on. The assembled Li/CPE-LCT/LiCoO_2_ coin cell can also deliver high initial discharge specific capacity and steady rate cycle performance. Those excellent results indicate that the as-prepared CPEs modified with the spherical-like and honeycomb structural Li_0.1_Ca_0.9_TiO_3_ particles can be developed as a new kind of practical polymer electrolyte.

## Author contributions

WX, PM, and JL contributed conception and design of the study. CM organized the database. XY and MT performed the statistical analysis. ZW wrote the first draft of the manuscript. XY, YZ, YJ, and MT wrote sections of the manuscript.

### Conflict of interest statement

The authors declare that the research was conducted in the absence of any commercial or financial relationships that could be construed as a potential conflict of interest.
